# Hydraulic (Single Cone) Versus Thermogenic (Warm Vertical Compaction) Obturation Techniques: A Systematic Review

**DOI:** 10.7759/cureus.62925

**Published:** 2024-06-22

**Authors:** Haytham S Jaha

**Affiliations:** 1 Restorative Dentistry, Umm Al-Qura University, Makkah, SAU

**Keywords:** cold lateral compaction (clc), warm vertical compaction (wvc), single cone (sc), continuous wave condensation (cwc), root canal treatment (rct)

## Abstract

Root canal treatment (RCT) involves cleaning and shaping of the root canal system before filling the canals with obturating materials, often gutta-percha (GP). The two primary obturation techniques are the thermogenic (warm vertical compaction, WVC) and hydraulic (single cone, SC) techniques. The objective of this study is to compare the clinical results and effectiveness of thermogenic and hydraulic obturation procedures in endodontic therapy to provide clinicians with evidence-based recommendations. Search strategies were conducted on February 01, 2024 and involved the databases Web of Science, PUBMED, Google Scholar, Scopus, Medline, Embase, NCBI, and Cochrane Library. The current systematic review included systematic reviews; meta-analyses; cohort studies; randomized controlled trial (RCT) studies; studies involving the comparison between single cone and warm compaction techniques; studies involving outcomes that include advantages, disadvantages, and complications associated with single cone or warm compaction techniques; and studies published within the last seven years. The selected studies were restricted to those with insufficient data, review articles without authentic references, publications in a language other than English, animal studies, and studies not involving SC or WVC. Out of 2300 studies registered, only 12 studies were included in the review. Both WVC and SC techniques showed satisfactory root canal fillings. However, five studies showed differences in the filling quality, four studies assessed the sealing ability and the dentinal tubule penetration of sealers, one study compared dentinal cracks, one study evaluated the amount of debris excluded apically, and one study compared the post-operative pain while using both techniques. The WVC and SC obturation techniques offer advantages and disadvantages in endodontic treatment. SC obturation is a simple and efficient procedure that is particularly suitable for situations with uncomplicated canal structures. The WVC obturation approach provides superior flexibility and sealing capability, especially in the complex root canal system. When choosing the method of treatment, it is important to take into account the patient's preferences, the clinician's experience, and unique considerations related to the situation. This systematic review highlights the important recommendations to healthcare professionals in selecting the most suitable obturation procedure based on the specific requirements of each clinical scenario. Research involving long-term follow-ups is required to get a better understanding of the outcomes of long-term goals. Clinical relevance: ability to educate clinicians regarding the best obturation technique between thermogenic and hydraulic. It directs the treatment decisions to maximize patient’s comfort, minimize post-operative complications, and improve efficacy in endodontic practice.

## Introduction and background

Apical periodontitis is an inflammatory reaction that occurs in the periapical tissues resulting from an infection in the root canal system. In such cases, root canal therapy is the treatment of choice [1]. Root canal treatment (RCT) aims to save the natural tooth by eradicating the underlying periapical infection [2]. Dental pulp inflammation, also known as pulpitis, is categorized into two types: reversible pulpitis (after managing the etiology, the inflammation should resolve and the pulp should revert to normal), and irreversible (signifying that the essential inflamed pulp is unable to undergo healing) pulpitis. Microbes are the major contributing factor to irreversible pulpitis. For irreversible pulpitis, it is advisable to have a conventional RCT that typically requires single or multiple visits to complete the procedure [3].

RCT is a common dental procedure performed by specialized endodontists and general dentists. The steps of RCT include pulp chamber opening, pulpectomy, chemo-mechanical disinfection of infected root canal system, and receiving obturation filling materials [4]. The main indications for RCT are irreversible pulpitis, apical periodontitis, periapical infection, complicated crown fractures, tooth necrosis due to trauma, and sub-crestal coronal third root fracture [5]. The purpose of endodontic diagnosis is to determine the pulpal and periradicular status and presence of associated disease based on clinical observations and tests so that the cases can be appropriately managed. The anatomically confined nature of the pulp and periradicular tissues makes direct observation, sampling, or testing to determine their health status difficult to perform. Therefore, a diagnostic process comprising assessments of the patient history, clinical signs and symptoms, sensibility tests, and radiological information is required for inferred verification of their health status. The American Association of Endodontists (AAE) standardizes the pulp diagnosis as normal, reversible pulpitis, symptomatic and asymptomatic irreversible pulpitis, and necrotic pulp. Sudden pain upon percussion and sensitivity to the cold test indicate symptomatic pulpitis. Meanwhile, little tenderness upon percussion, no sharp pain, and no radiographic radiolucency indicate asymptomatic irreversible pulpitis. Necrotic pulp is characterized by no sensitive response in the cold test [6].

Successful RCT depends on proper canal irrigation, shaping, and obturation. An optimal root canal irrigation procedure should effectively eliminate germs, biofilm, and smear layer, while thoroughly disinfecting all components of the root canal system. The primary goal of proper canal shaping is to preserve or enhance a consistently narrowing funnel shape from the canal opening to the tip. Obturation is considered the crucial and final step, which involves filling the root canal. Improper or inadequate filling can lead to periapical reinfection. Proper obturation without voids is a significant factor for successful RCT [7]. 

The obturating materials used in primary teeth differ from those used in permanent teeth. Zinc oxide-eugenol paste is the first material of choice for the obturation of primary teeth [8]. During pulpectomy in primary teeth, there is a risk of damaging the underlying permanent dentition. Additionally, the treatment can be influenced by various factors, such as the root curve. Therefore, the obturating material of choice and technique in primary dentition differ from those in permanent dentition. Zinc oxide-eugenol, calcium hydroxide, and iodoform-based pastes are the most commonly used materials, and techniques involve the use of endodontic pressure syringes, Lentulo spirals, and endodontic pluggers/reamers [9].

Proper obturating materials and techniques are required for three-dimensional stable root canal filling [10]. A three-dimensional and homogenous filling can lead to successful RCT, while voids or unfilled spaces of the canal can lead to complications and treatment failure. Additionally, sealers are important in creating an adequate seal in the canal. Various sealers expand on the setting, thereby filling the spaces. They also contain an antimicrobial agent that reduces infection. Such sealers include calcium-based Endosequence BC (Brasseler, Savannah, GA, USA) [11].

There are several obturation techniques to perform RCT, such as the cold lateral condensation technique, single cone (SC-hydraulic) technique, and injectable thermoplasticized gutta-percha (GP) technique (continuous or interrupted) [12]. The cold lateral compaction (CLC) technique is the most simple and widely used. A previous study showed that the CLC technique is the gold standard, while the warm lateral compaction technique is preferred by participants [13]. However, it has major disadvantages, including vertical root fracture, due to applying extra hard compaction forces.

In the 1967s, the warm vertical compaction (WVC) technique was introduced by Schilder with the aim of enhancing root canal-wall adaptation [14]. The WVC technique is the preferred choice in endodontics. The WVC technique includes heating the GP until it softens and alters its phase, thereby adapting to the wall of the prepared root canal. In this technique, less sealer and more heated GP are used, allowing for the use of any sealer brand [15,16]. The WVC technique is associated with better adaptation to the canal wall. However, this technique is time-consuming, responsible for the sealer’s apical extrusion, leading to post-operative pain [17-19]. With practice, GP control has improved, reducing operating time and benefiting from the heated GP approach [20]. Generally, the WVC technique needs a lower proportion of sealer than the cold filling technique.

Recently, the SC obturation technique has gained popularity in recent years. The SC technique comprises the preparation of the canal with wider taper instruments and filling the canal with the matched GP; therefore, it is less technique-sensitive and much easier and became better alternative [21]. In the SC technique, a tapered GP cone matching the size of the final prepared canal is inserted into the canal with a sealer, without needing an accessory GP cone [22]. However, some complications still exist with the SC technique, such as the presence of voids when used with the AH-plus epoxy resin sealer (Dentsply Sirona, Konstanz, Germany) and active-GP glass ionomer sealer [23,24]. Micro-leakage can be avoided by ensuring homogenous contact among the sealer, GP, and dentin [25]. Inadequate filling of the root canal is a major cause of RCT failure. A tug-back is recommended in the SC technique by the master GP cone for a proper seal [26]. The SC technique is associated with the least extrusion of material beyond the apex but has poor and uneven lateral wall adaptation [27].

With SC and WVC being the most commonly used techniques, this review aimed to compare the clinical results and effectiveness of thermogenic and hydraulic obturation procedures in endodontic therapy to provide clinicians evidence-based recommendations.

## Review

Methods

Search Strategy

This systematic review was built upon a meticulous adherence to the guidelines set forth by the Preferred Reporting Items for Systematic Reviews and Meta-Analyses (PRISMA) guidelines in 2020, ensuring a stringent commitment to transparency and comprehensiveness [28]. The PRISMA checklist is given in Table 2 of Appendix. Search strategies on the databases Web of Science, PUBMED, Google Scholar, Scopus, Medline, Embase, NCBI, and Cochrane Library were performed on February 1, 2024. The search key terms included "single cone obturation technique," "warm vertical compaction technique," "thermoplasticized gutta-percha," and "root canal filling materials" to combine these terms effectively. The search strategy was tailored for each database; for example, in PubMed, the search string was "single cone obturation" OR "warm obturation technique" AND " root canal filling materials." When appropriate, the full text of the articles was reviewed.

Selection Criteria

The inclusion criteria of the current study were (1) systematic reviews, meta-analysis, cohort studies, randomized controlled trial (RCT) studies; (2) studies involving the comparison between SC and warm compaction techniques; (3) studies involving outcomes that include advantages, disadvantages, and complications associated with SC or warm compaction techniques; and (4) studies published within the last seven years. The screening was restricted to (1) studies with insufficient data, (2) review articles with no authentic references, (3) publications in a language other than English, (4) animal studies, and (5) studies not involving the SC or WVC techniques.

Study Analysis

**Figure 1:**
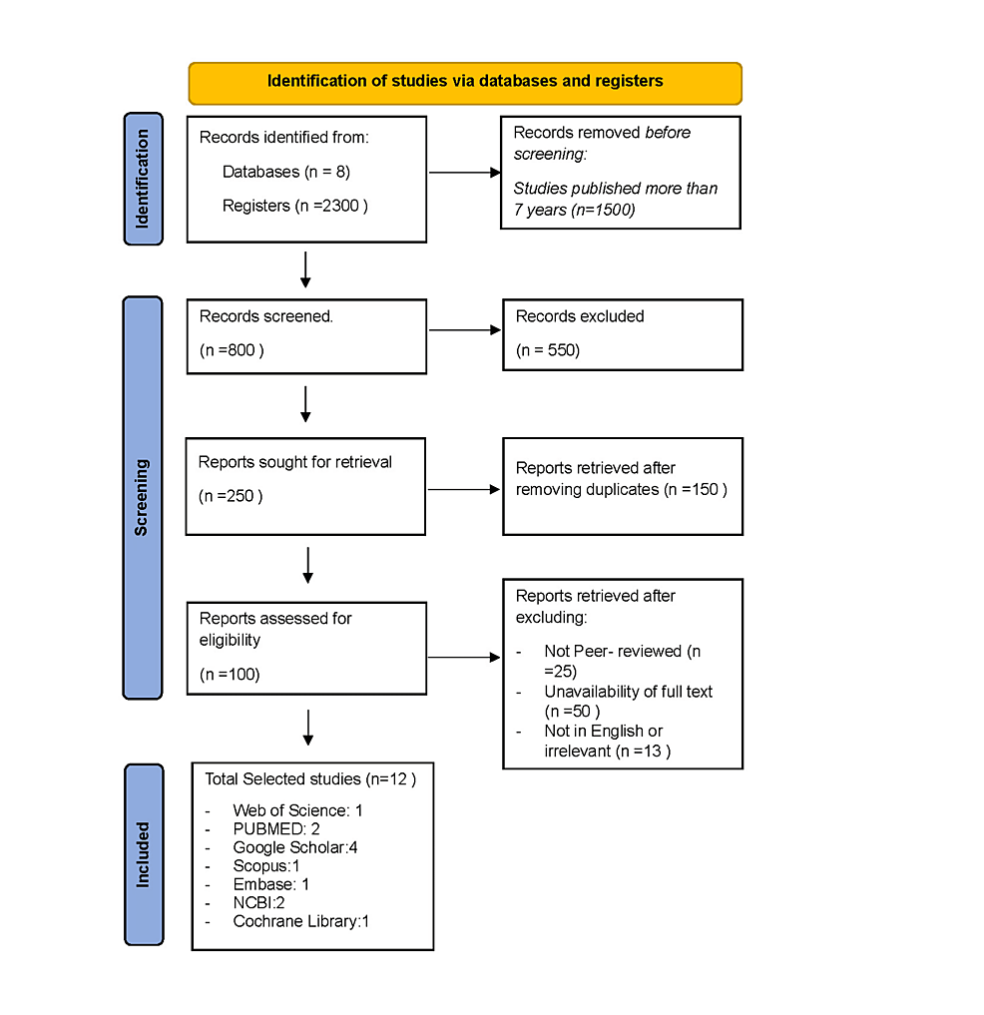
PRISMA flowchart of the process

The objective of this study was to incorporate reliable, recent, and up-to-date research on hydraulic and WVC techniques. The databases include Google Scholar, PUBMED, Scopus, Web of Science, NCBI, Medline, Embase, and Cochrane Library. 2300 studies were gathered. In contemporary dentistry, this study examines obturation techniques and their importance and outcomes that improve patients’ oral health. The primary goal is to thoroughly analyze the data, literature, and findings from earlier research projects. With the help of the keywords, almost 800 studies from 2017 to 2024 were found. The terms hydraulic and thermogenic obturation were used to filter these studies further and 250 were returned. Using the more focused keywords, SC obturation technique, and warm compaction technique, these papers were further refined and underwent exclusion criteria. After assessment, irrelevant, unsuitable, and unfit studies were excluded, and the number of studies was reduced to 100. Among these, some research was published in a language other than English, and some were not subjected to peer review and total 12 studies included in all. The steps are displayed in Figure 1.

Data Extraction

The reviewer effectively extracted and analyzed data from the full texts of the selected articles, covering various aspects such as general information, introduction, study location, criteria for selecting the quality management system, and authorities used in the study, analytical methods, type of guidelines, discussion of data conclusions, future perspectives, and limitations of the studies. The meta-analysis was conducted following data extraction and qualitative synthesis of individual study findings. If deemed appropriate based on homogeneity of included studies and outcome measures, a quantitative synthesis or meta-analysis was performed using statistical software. This involved pooling effect estimates from individual studies to generate an overall summary estimate of treatment effect, with measures of statistical heterogeneity assessed to determine result consistency and sensitivity analyses conducted to explore potential bias sources. Table 1 summarizes the 12 study matrices to illustrate the main findings and aims of the included studies in this review.

**Table 1:** Description of studies selected for systematic review SC, single cone; RCT, randomized controlled trial; WVC, warm vertical compaction; CLC, cold lateral compaction; GP, gutta-percha

Name and year of the study	Study design	Sample size	Purpose	Outcome
Iglecias et al., 2017 [17]	Micro-computed tomography analysis	n=24, mandibular molars	Comparison of the presence of voids in the canal that had been obturated with SC and continuous wave of condensation	Compared to SC, the CWC technique produced fewer voids in the cervical third
Donnermeyer et al., 2018 [20]	Experimental study	N=28, single rooted maxillary canine	To evaluate the temperature raise in endodontic sealers during obturation techniques in a closed system	Continuous wave technique: increased temperature. Than thermafil and SC obturation
Canakci et al., 2019 [18]	Experimental study	n=100, human incisor teeth	Amount of debris extruded apically during retreatment	Debri extrusion: WVC>CLC
Aydinbelge et al., 2019 [19]	Comparative Experimental Study	n=55, mandibular teeth with mature apices and straight canal	Comparison of dentinal cracks after cold lateral, WVC, and SC technique	Dentinal cracks: CL and WVC >SC
Moccia et al., 2020 [14]	Micro-CT study	n=30, single-rooted human teeth	The aim is to assess the 3D filling by WVC, SC, and carrier-based	Specifically, bioceramic sealer with SC technique proved a practical option, even though this method seemed more dependent on the operator’s skill
Dasari et al., 2020 [15]	Comparative experimental study	n=90, mandibular premolar teeth	Evaluate the penetration depth and root dentin adaptation of bioceramic root canal sealer using WVC and lateral condensation technique	WVC technique showed more sealer penetration and fewer coronal, middle, and apical gaps. Meanwhile, lateral condensation showed more gaps and less sealer penetration
Bhagat et al., 2021 [29]	Invitro experimental study	n=30, simulated, 30-degree curve root canal transparent acrylic blocks.	Comparison of CL and WVC technique using continuous wave of compaction	WVC GP filling>CL
Eid et al., 2021 [11]	Invitro experimental study	n=44, mandibular human premolars (single rooted)	Evaluate the dentinal tubule penetration of two calcium silicate-based sealers between WVC and SC	Compared to the SC approach, WVC improved the penetration of calcium silicate-based sealers into the dentinal tubules
Pontoriero et al., 2021 [30]	RCT	n=80, maxillary human premolars	Evaluate Bioceramic and ZOE sealer’s sealing ability with SC and WVC techniques	The sealers are suitable to use with both SC and WVC techniques
Zhang et al., 2021 [31]	Micro-computed tomographic study	n=24, artificial molar teeth with band-shaped isthmuses	Presence of spaces in band-shaped isthmuses using LC, SC, and CWC techniques	The obturation quality of CWC was better than LC and SC
Bugea et al., 2022 [32]	RCT	n=40, teeth	Compare the post-operative pain after one week and the success rate after one-year follow-up in patients who underwent SC and WVC techniques	WVC group: 10/10 pain to percussion. SC group: 2/10 pain to percussion Bioconeless: 1/10 Pain to percussion coneless: 9/10 pain to percussion
Vasconcelos et al., 2022 [13]	Cross-sectional survey		To assess the current obturation trends including the use of calcium silicate-based sealers and preferred use of warm vs cold obturation technique	Users of WOT: 58.7%. COT: 41.3%. Most selected sealer: epoxy resin-based sealer: 52.3%. Training in endodontics and working situation impact the selection of warm or cold obturation technique (p<0.001)

Results

The combined outcomes showed that the success rates of the thermogenic (WVC) and hydraulic (SC obturation technique) obturation methods were similar. WVC techniques seemed to give advantages in terms of better adaptability to root canal walls and fewer voids compared to hydraulic approaches, while both methods indicated appropriate root canal fills. For both approaches, the rates of complications were generally low. The review’s strong points were a thorough search technique, analysis of authentic papers, and synthesizing results from various sources. WVC and SC techniques should be compared using standardized protocols in the future. The protocols should include consistent outcome assessments and longer-term follow-ups to evaluate the clinical importance and durability of any identified outcome variations.

Although both techniques showed satisfactory root canal fillings, certain investigations found differences in filling quality between the thermogenic and hydraulic techniques. Thermogenic techniques seemed to have an advantage in better canal wall adaptation and fewer spaces than hydraulic techniques. Meanwhile, the SC technique is the advanced technique that saves time and is cost-effective; only a single master cone that is similar to the size of the prepared canal is required. Also, it causes less pressure on the canal walls; therefore, the chances of root fracture are reduced, which is a major disadvantage of the lateral compaction obturation technique. 

This systematic review raises the possibility that more uniform and well-suited root canal fillings could be produced using WVC or SC technique, which could improve the treatment results. When choosing the best obturation technique for a patient, dental practitioners should consider operator experience, treatment objectives, and root canal anatomy.

Discussion

The complex endodontic system consists of accessible and non-accessible areas. Accessible areas include the pulp chamber and the main canals, and manual and rotary files are easily reached and prepared. Also, some places are inaccessible, including dentinal tubules and lateral canals. Mechanical shaping cannot reach every area of this complex anatomy, leaving certain canals untreated. For this reason, endodontic biochemical cleaning is essential, and it is sealed during the obturation process using GP and a sealer [33].

The two main studies discussed in various papers are CLC and WVC. According to one prospective clinical study, the WVC technique for obturating the canals showed better results and outcomes than CLC in preoperative periapical lesions [34] 

Sealers, such as Bioceramic used during obturating canals, behave as an adhesive between the GP cone and the canal. Bioceramic sealer penetrates deep into the dentinal tubule, creating a strong mechanical bond between the GP cone and the canal [35].

The most common root canal filling material used in the RCT is the GP due to its dimensional stability, biocompatibility, and natural plasticity when applied heat. Also, it can be easily removed from the canal when reendodontic treatment is required [36]. Post-operative pain is common after RCT. It occurs due to applying pressure and apical instrumentation during cleaning and shaping, which leads to the extrusion of the debris. The risk of pain increases when there is an underlying periapical inflammation. A single-visit root canal is preferred, which reduces the chances of reinfection [37]. Single-visit root canals have many advantages over multiple-visit root canals. It includes time-saving and the least chances of bacterial contamination and reinfection. Therefore, single-visit RCTs are preferred by dental practitioners [38].

One of the most common causes of endodontic failure is the coronal leakage. The procedure is improved using improved quality, bioceramic sealers, and better obturation techniques. Dental practitioners involve SC or hydraulic condensation techniques [39]. With advancements in dentistry, complex treatments are becoming simpler and more effective. SC technique is considered to be much easier than other endodontic obturation techniques. It showed a sealing ability similar to that of different techniques. Better quality sealers are also important in successful RCTs; one is AH Plus (Dentsply Maillefer Ballaigues, Switzerland) [40].

Spaces between the filling material and the walls of the canal can undermine the success of the endodontic treatment, allowing bacteria to move into the spaces and apical foramen, leading to the formation of apical periodontitis. Obturation technique should be capable of adaptation of the filling material, covering the whole length of the canal wall [41].

Different types of sealers have been suggested to fill the voids between the GP and the canal walls. To decrease gaps, the sealers must build a tight and adequate seal between the core material and the dentine. A 3D obturation is likely to result in the fluid tight seal to overcome micro-leakage. The deep penetration of the sealer into the dentinal tubules helps in the formation of a sufficient seal to entrap leftover germs [11-16].

The foundation of successful RCT is the diagnosis, treatment planning, knowledge of dental anatomy, and application of the best root canal and obturation technique possible. Better access to the canal provides quality obturation. Therefore, along with the obturation technique, dental professionals also recommend focusing on proper cleaning and shaping. Root canal therapy is usually successful if the canal is properly cleaned and shaped [42].

An observational study was conducted to get information regarding the current and updated status of the preferred obturation technique and the choice of materials. The study showed that WVC technique users made up 58.7% of the total, while cold obturation technique users made up 41.3% [13].

The RCT study compared the micro-leakage of different obturation techniques after post-space preparation with other drills. Peeso drills showed safer results among all obturation techniques. Meanwhile, Gates-Glidden drills caused increased leakage when used with the SC technique. More literature review is required to better understand the comparison [43]. Very little data on the sealing ability of the SC and WVC techniques is present. Studies discussing long-term reactions of the underlying periapical tissues will help provide better knowledge. In a recent survey, the sealing property of the SC technique is more or less similar to the WVC technique [44].

The success rate of RCT depends upon properly cleaning and shaping the canal walls with a three-dimensional filling of the root canal. Therefore, an adequate obturation technique is essential in the root canal procedure [45]. Periapical radiographs are also used to evaluate the filling of the root canal more clearly. Homogenous and complete filling of the root canal up to the level of working length of the canal leads to the successful RCT. The two-dimensional radiographic image such as periapical radiograph does not show the full adaptation of the filling material to the canal walls [46].

## Conclusions

Although success and combination rates for hydraulic and thermogenic obturation techniques are generally comparable, the heterogeneity in study designs and outcomes highlights the necessity for standardization in future research. Long-term follow-up and uniform outcome assessments would improve the findings. There are benefits and drawbacks to both WVC and SC (hydraulic) obturation procedures in endodontic therapy. Hydraulic obturation is easy to use and effective, and it is a good option for common instances involving simple canal anatomy. However, thermogenic obturation offers better adaptability and sealing capacity, especially in the complex endodontic system involving complicated tooth canals. Patient preferences, clinician experience, and specific case factors should all be considered when selecting the two methods. This systematic review offers helpful guidelines to clinicians in choosing the best obturation technique according to the particular needs of every clinical situation.

## Appendices

**Table 2:** Prisma checklist 2020 Page MJ, McKenzie JE, Bossuyt PM, Boutron I, Hoffmann TC, Mulrow CD, et al. The PRISMA 2020 statement: an updated guideline for reporting systematic reviews. BMJ 2021;372:n71. doi: 10.1136/bmj.n71 For more information, visit: http://www.prisma-statement.org/

	Item #	Checklist item	Location where item is reported
TITLE			
Title	1	Identify the report as a systematic review.	1-2
ABSTRACT			
Abstract	2	See the PRISMA 2020 for abstracts checklist.	26-54
INTRODUCTION			
Rationale	3	Describe the rationale for the review in the context of existing knowledge.	56-154
Objectives	4	Provide an explicit statement of the objective(s) or question(s) the review addresses.	150-4
METHODS			
Eligibility criteria	5	Specify the inclusion and exclusion criteria for the review and how studies were grouped for the syntheses.	162-77
Information sources	6	Specify all databases, registers, websites, organizations, reference lists, and other sources searched or consulted to identify studies. Specify the date when each source was last searched or consulted.	178-91
Search strategy	7	Present the full search strategies for all databases, registers, and websites, including any filters and limits used.	155-91
Selection process	8	Specify the methods used to decide whether a study met the inclusion criteria of the review, including how many reviewers screened each record and each report retrieved, whether they worked independently, and if applicable, details of automation tools used in the process.	178-92
Data collection process	9	Specify the methods used to collect data from reports, including how many reviewers collected data from each report, whether they worked independently, any processes for obtaining or confirming data from study investigators, and if applicable, details of automation tools used in the process.	-
Data items	10a	List and define all outcomes for which data were sought. Specify whether all results that were compatible with each outcome domain in each study were sought (e.g., for all measures, time points, analyses), and if not, the methods used to decide which results to collect.	-
	10b	List and define all other variables for which data were sought (e.g., participant and intervention characteristics, funding sources). Describe any assumptions made about any missing or unclear information.	-
Study risk of bias assessment	11	Specify the methods used to assess risk of bias in the included studies, including details of the tool(s) used, how many reviewers assessed each study, and whether they worked independently, and if applicable, details of automation tools used in the process.	
Effect measures	12	Specify for each outcome the effect measure(s) (e.g., risk ratio, mean difference) used in the synthesis or presentation of results.	--
Synthesis methods	13a	Describe the processes used to decide which studies were eligible for each synthesis (e.g., tabulating the study intervention characteristics and comparing against the planned groups for each synthesis (item #5)).	162-77
	13b	Describe any methods required to prepare the data for presentation or synthesis, such as handling of missing summary statistics or data conversions.	-
	13c	Describe any methods used to tabulate or visually display results of individual studies and syntheses.	-
	13d	Describe any methods used to synthesize results and provide a rationale for the choice(s). If meta-analysis was performed, describe the model(s) and method(s) to identify the presence and extent of statistical heterogeneity and software package(s) used.	423-4
	13e	Describe any methods used to explore possible causes of heterogeneity among study results (e.g., subgroup analysis, meta-regression).	-
	13f	Describe any sensitivity analyses conducted to assess robustness of the synthesized results.	-
Reporting bias assessment	14	Describe any methods used to assess risk of bias due to missing results in a synthesis (arising from reporting biases).	-
Certainty assessment	15	Describe any methods used to assess certainty (or confidence) in the body of evidence for an outcome.	-
RESULTS			
Study selection	16a	Describe the results of the search and selection process, from the number of records identified in the search to the number of studies included in the review, ideally using a flow diagram.	429-6
	16b	Cite studies that might appear to meet the inclusion criteria, but which were excluded, and explain why they were excluded.	
Study characteristics	17	Cite each included study and present its characteristics.	423-4
Risk of bias in studies	18	Present assessments of risk of bias for each included study.	-
Results of individual studies	19	For all outcomes, present, for each study: (a) summary statistics for each group (where appropriate) and (b) an effect estimate and its precision (e.g., confidence/credible interval), ideally using structured tables or plots.	-
Results of syntheses	20a	For each synthesis, briefly summarize the characteristics and risk of bias among contributing studies.	-
	20b	Present results of all statistical syntheses conducted. If meta-analysis was done, present for each the summary estimate and its precision (e.g., confidence/credible interval) and measures of statistical heterogeneity. If comparing groups, describe the direction of the effect.	-
	20c	Present results of all investigations of possible causes of heterogeneity among study results.	194-214
	20d	Present results of all sensitivity analyses conducted to assess the robustness of the synthesized results.	-
Reporting biases	21	Present assessments of risk of bias due to missing results (arising from reporting biases) for each synthesis assessed.	-
Certainty of evidence	22	Present assessments of certainty (or confidence) in the body of evidence for each outcome assessed.	-
DISCUSSION			
Discussion	23a	Provide a general interpretation of the results in the context of other evidence.	194-214
	23b	Discuss any limitations of the evidence included in the review.	237-43
	23c	Discuss any limitations of the review processes used.	237-48
	23d	Discuss implications of the results for practice, policy, and future research.	268-71
OTHER INFORMATION			
Registration and protocol	24a	Provide registration information for the review, including register name and registration number, or state that the review was not registered.	-
	24b	Indicate where the review protocol can be accessed or state that a protocol was not prepared.	-
	24c	Describe and explain any amendments to information provided at registration or in the protocol.	-
Support	25	Describe sources of financial or non-financial support for the review and the role of the funders or sponsors in the review.	-
Competing interests	26	Declare any competing interests of review authors.	287-8
Availability of data, code, and other materials	27	Report which of the following are publicly available and where they can be found: template data collection forms; data extracted from included studies; data used for all analyses; analytic code; any other materials used in the review.	-
